# Neural correlates of rules and conflict in medial prefrontal cortex during decision and feedback epochs

**DOI:** 10.3389/fnbeh.2015.00266

**Published:** 2015-10-06

**Authors:** Gregory B. Bissonette, Matthew R. Roesch

**Affiliations:** ^1^Department of Psychology, University of Maryland, College ParkCollege Park, MD, USA; ^2^Program in Neuroscience and Cognitive Science, University of Maryland, College ParkCollege Park, MD, USA

**Keywords:** set-shifting, mPFC, rule encoding, conflict, *in vivo* electrophysiology

## Abstract

The ability to properly adjust behavioral responses to cues in a changing environment is crucial for survival. Activity in the medial Prefrontal Cortex (mPFC) is thought to both represent rules to guide behavior as well as detect and resolve conflicts between rules in changing contingencies. However, while lesion and pharmacological studies have supported a crucial role for mPFC in this type of set-shifting, an understanding of how mPFC represents current rules or detects and resolves conflict between different rules is unclear. Here, we directly address the role of rat mPFC in shifting rule based behavioral strategies using a novel behavioral task designed to tease apart neural signatures of rules, conflict and direction. We demonstrate that activity of single neurons in rat mPFC represent distinct rules. Further, we show increased firing on high conflict trials in a separate population of mPFC neurons. Reduced firing in both populations of neurons was associated with poor performance. Moreover, activity in both populations increased and decreased firing during the outcome epoch when reward was and was not delivered on correct and incorrect trials, respectively. In addition, outcome firing was modulated by the current rule and the degree of conflict associated with the previous decision. These results promote a greater understanding of the role that mPFC plays in switching between rules, signaling both rule and conflict to promote improved behavioral performance.

## Introduction

The inability to alter behavioral responding in order to adapt behavior to changing situations is a hallmark of many human psychiatric disorders (Gold et al., 2008, 2009; Strauss et al., 2011). Patients who suffer from deficits in flexible behavior are able to learn information and form rules which instruct and guide choices, but lack the ability to alter their choices when contingencies change (Cools et al., 2000; Shamay-Tsoory et al., 2004). Appropriate use of behavior-guiding rules can lead to effective behavioral flexibility, enabling animals to successfully navigate an ever changing world (Harlow, 1949; Roesch et al., 2010). Patients with many disorders, including schizophrenia (Elliott et al., 1995; Pantelis et al., 1999), Parkinson’s disease (Gauntlett-Gilbert et al., 1999; Monchi et al., 2004; Dirnberger and Jahanshahi, 2013) or drug addiction (Lyvers and Yakimoff, 2003) struggle with this ability, as studied on the Wisconsin Card Sorting Task (WCST). The WCST requires individuals to discriminate relevant from irrelevant information while sorting cards based on color, shape or number (Nelson, 1976; Prentice et al., 2008). Patients with the aforementioned disorders can, for example, sort cards by shape, and ignore irrelevant features like color and number, but when the sorting rule changes, they struggle to sort by number and ignore color and shape.

The animal literature clearly indicates medial prefrontal cortex (mPFC) is critical for some aspect of attentional set-shifting (Dias et al., 1996a,b; Birrell and Brown, 2000; Colacicco et al., 2002; Bissonette et al., 2008; Roy et al., 2010), a function captured in the shift between sorting parameters during the WCST. Few studies have attempted to record from single neurons in mPFC while animals learned or shifted between rule strategies, and the majority of these studies occur in primates (White and Wise, 1999; Wallis et al., 2001; Bunge et al., 2003; Muhammad et al., 2006; Durstewitz et al., 2010). Yet it is not clear how these representations in mPFC develop, or how they ultimately guide behavior via downstream behavioral circuits.

Interference work has shown us that mPFC plays some role in forming associations between stimuli, responses, and outcomes so that one can learn the contingencies necessary to perform these types of tasks (Boettiger and D’Esposito, 2005; Oliveira et al., 2007). Rat dorsal mPFC (mainly pre-limbic cortex) mediates spatial working memory and visual object information, along with cross-modal switching involving spatial location, visual objects and spatial locations with motor responses (Seamans et al., 1995; Kesner et al., 1996; Ragozzino et al., 1998, 1999a,b). Although mPFC lesions impact set-shifting, they do not impair initial learning (Dias et al., 1996a; Birrell and Brown, 2000; Bissonette et al., 2008), suggesting that mPFC is not essential for rule learning, but is critical when rule contingencies change. Such a deficit might reflect a misrepresentation of rules after shifts and/or the inability to detect errors and resolve conflict between competing rules. Consistent with these hypotheses, neurophysiological work in behaving animals has shown us that activity in mPFC encodes expected value, future actions, stimulus-response associations, and is spatially selective (Nieder et al., 2002; Horst and Laubach, 2009, 2012; Narayanan and Laubach, 2009; Balleine and O’Doherty, 2010). Further, neural ensemble firing in mPFC reflects distinct active states during set-shifting, which is temporally related to behavioral performance (Durstewitz et al., 2010; Roy et al., 2010; Antzoulatos and Miller, 2011).

Here we furthered the investigation of mPFC’s role in attentional set-shifting by recording in rats as they performed a two direction set-shifting task during which behavior is guided by odors or spatial cue lights. We found that a subset of mPFC neurons fired more strongly for one rule over another, and that activity in this population of neurons briefly increases early in a rule block, possibly signaling a need for shifting between rule-based strategies. Interestingly, a separate population of neurons represented both the response direction and the conflict inherent in the task, firing more for high conflict, low certainty trials, over low conflict, more certain trials. Finally, we show that all of these neural subtypes multiplex information by encoding both rewarded and non-rewarded outcomes differently. Together, these data suggest that some mPFC neurons encode one rule preferentially over another while other mPFC neurons are more active during high conflict trials during decision and feedback epochs, further supporting a role for increased attention signals in mPFC and showing how these attention signals mediate mPFC rule encoding.

## Materials and Methods

### Subjects

Five single housed adult male Long-Evans rats (175–200 g) obtained from Charles River Labs (Wilmington, Massachusetts) and were tested at the University of Maryland, College Park, in accordance with the university and National Institutes of Health guidelines and with approval from University of Maryland, College Park Institutional Animal Care and Use Committee.

### Set-Shifting Task and Analysis

**Figure 1:**
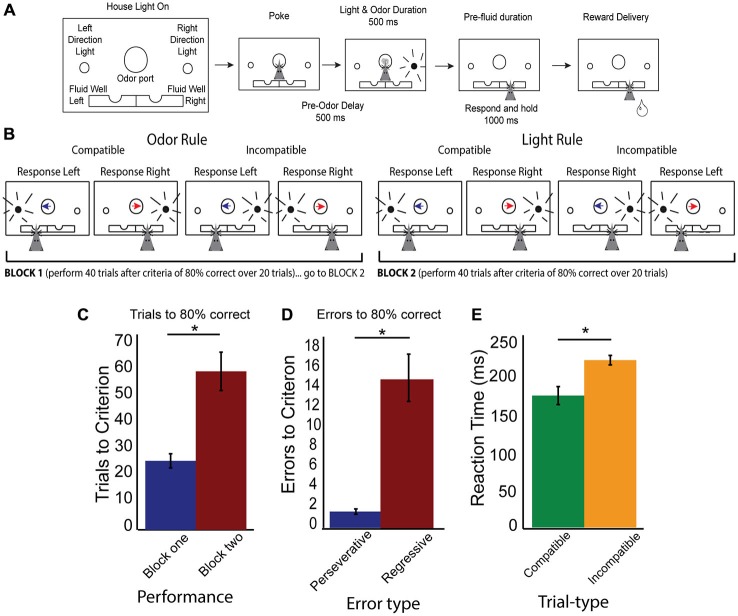
**Rule shifting behavior schematic and measures. (A)** Schematic of odor port, fluid wells and simultaneous directional cues as well as flow chart of successful trial completion denoting specific time delays. **(B)** Example correct responses within different rule blocks, demonstrating compatible and incompatible trials with simultaneous directional information presented by both cues. Blue arrow indicates the “left” direction odor, Red arrow indicates the “right” direction odor, while black radiating circle represents the side of the light. Rat head represents the correct choice made by a rat which would have previously received the simultaneous presentation of both odor and light cues before making his choice. **(C)** Data showing increased number of trials required to reach criterion after a switched rule, with trials to criterion in block one (blue bar) and block two (red bar). **(D)** Data demonstrating a significant number of errors are regressive (red bar), rather than perseverative (blue bar). **(E)** Reaction time data (cue offset to port exit), demonstrating significant slowing on incompatible (orange bars) compared to compatible trial-types (green bars). Significance of at least *p* < 0.05 denoted with asterisk *.

Rats were required to nosepoke and follow a cue to a well for a fluid reward (10% sucrose). To train rats to nosepoke, wait the required delay periods (500 ms pre-cue, 500 ms cue, 1000 ms pre-fluid delay) and respond to fluid wells for reward took approximately 2 weeks. Rats were then trained to respond to left or right direction lights for reward (approximately 2 weeks of training) and to respond left or right for two distinct olfactory cues (approximately 2 weeks of training). Once rats were proficient at both light and odor responses, rats were given a week of training where odors and lights were presented together, yet on any given day, only one “rule” was to be followed (e.g., on Monday, follow lights and ignore odors, on Tuesday, follow odors and ignore lights). Once rats were performing over an 80% success rate on both a light and odor rule day, they underwent surgical implantation of recording electrodes. The recording chamber is identical to those used in previous work (Roesch et al., 2007; Roesch and Bryden, 2011). One wall panel has a central odor port (1″ wide) with cue lights (EiKO 20.11 lumen bulbs) placed 3″ on either side of the odor port, such that rats, when fully nosepoking, can still see a light to their left or right. One and three fourths inches below the odor port and $1^{\frac{1}{4}}$” to both the left and right side of the odor port are the fluid wells. The cartoon representation in Figures 1A,B which also provides trial-type information gives an approximate representation of the size and position. For photos of the odor panel, please see Roesch et al. (2007), Roesch and Bryden (2011).

During the set-shifting task, we combined the presentation of both cues so that animals received simultaneous light and odor information (Figure 1A). When a house light was illuminated, rats were required to nosepoke and hold for 500 ms in order to receive 500 ms of simultaneous and random odor and direction light pairing. Rats were required to wait the entire 500 ms duration of the cues, then to respond to a direction of one of the dimensions of cue (odor or light), but not the other. Once a response was made, rats were required to hold in the fluid well for 1000 ms, before the outcome was presented (either sucrose reward, or not). After a rat exited the fluid well after consumption of reward, the houselights went dark and the rats experienced a 5 s inter-trial interval (ITI). In the event of an error, once rats exited the fluid well an additional 3 s penalty was added to the 5 s ITI.

There were two main trial types (Figure 1B): trials where both cues indicated the same direction (e.g., right light and right odor) labeled as compatible trials, and incompatible trials, where both cues’ directions were in conflict with each other (e.g., right light and left odor). Rats began a day with the correct rule being the same as the final rule from the previous day and were to shift to the other rule in block two. Rules were counterbalanced daily. Performance was monitored by a running average of 20 trials. Once rats reached 80% correct in block one, an additional 40 trials were added onto their total before rules were shifted to avoid rats anticipating a rule shift and to collect enough data post-criteria from each trial-type analyze. In addition to collecting trials to criterion, we classified error types are perseverative or regressive, where a perseverative error was continued responding to the incorrect rule on incompatible trial-types after the switch and before any correct new rule trials while regressive errors were errors on incompatible trial-types after the first correct response in the new rule block, but before the rat reached criterion.

### Surgical Procedures

All surgical procedures were performed after training on the task. Five rats had a drivable bundle of 10, 25 μm diameter FeNiCr (iron, nickel, chromium) wires chronically implanted in the left or right hemisphere in dorsal mPFC at the top of prelimbic cortex (3.3 mm anterior to bregma, ±0.6 mm laterally, and 3.0 mm ventral to the brain surface) (Figure 1C; Bryden et al., 2012; Burton et al., 2013). After testing, rats were transcardially perfused with buffered 4% paraformaldehyde with brains postfixed at 4°C. Freezing microtome sections (50 μm) were cut and stained with Thionin. Cannula locations and electrode placements were verified under light microscope and drawn onto plates adapted from the rat brain atlas (Paxinos and Watson, 2004). If electrodes had been implanted into the wrong areas, rats would have been excluded from the study, though none were.

### Data Acquisition and Analysis

Experiments were performed in a behavioral chamber previously described (Schoenbaum and Roesch, 2005). We performed daily screening of active wires, and advanced the electrode assembly by ~80 μm per day at the end of the recording session to record from a different neuronal population. Neural activity was recorded using Plexon Multichannel Acquisition Processor systems (Dallas, TX). Signals from the electrode wires were amplified 20 times by an op-amp headstage (Plexon, HST/8o50-G20-GR), located on the electrode array. Immediately outside the chamber, signals were passed through a differential pre-amplifier (Plexon, PBX2/16sp-r-G50/16fp-G50), where the single unit signals were amplified 50 times and filtered at 150–9000 Hz. The single unit signals were then sent to the Multichannel Acquisition Processor box, where they were further filtered at 250–8000 Hz, digitized at 40 kHz and amplified at 1–32 times. Waveforms >2.5:1 signal-to-noise were extracted from active channels and recorded to disk. Neurons were sorted using Offline Sorter and Neuroexplorer (Burton et al., 2013), and exported for analysis in Matlab (Bissonette et al., 2013).

We used a least-squares multiple regression to determine the number of cells where firing rate was significantly correlated with either the response direction or rule block when variance for the two remaining factors was accounted for. To achieve this, we compared a base model (*k* = 2; where *k* = the number of parameters) to a complex model (*k* = 3) in two separate iterations, where Y = firing rate (spikes/s) during the 500 ms cue epoch, *Rule* = coded as (−1 = odor rule) (1 = light rule), *Direction* = coded as (−1 = right) (1 = left), *Compatibility* = coded as (−1 = compatible) (1 = incompatible).

We began by finding cells for which a single factor model led to significant change in neural activity. These neurons were grouped according to regressor. Following this, we calculated the number of cells that provided a significant improvement of fit (via Incremental F-test) when the second parameter in model 2 was added to the single factor model. Counts of correlated cells were compared via chi-square (*p* < 0.05). When analyzing neural activity during behavior, mean firing rate during cue epoch for populations of neurons was compared via ANOVA, with *post hoc t*-tests when appropriate. Data were checked for normal distribution by KS test and chi-square goodness of fit using Matlab functions *kstest* and *chi2gof*. Behavior results were analyzed with two-way ANOVA or one-way ANOVA with *post hoc t*-tests when appropriate or planned *t*-test. Means and Standard Error of the Mean (SEM) are provided for *t*-tests as well.

## Results

Rats readily learned to discriminate sensory cues and follow the appropriate rule. In 51 total sessions (out of 86), rats reached criterion in both the first and second rule blocks. Figure 1C demonstrates that shifting from rule one to rule two required significantly more trials and was challenging for the rats (*t*-test, Rule Means 26.3 vs 60.3, SEM 5.5 and 14.8, respectively, *p* < 0.001 *t* = −5.3, df = 50, Cohen’s *d* = −1, effect size = −0.44). When analyzing errors (Figure 1D) in behavior during the rule shift, we observed that the majority of errors were of the regressive type, as compared to perseverative (*t*-test, Means 1.2 and 15.6, SEM 0.5 and 4.8, *p* < 0.001, *t* = −6, df = 50, Cohen’s *d* = −1.32, effect size = −0.6: Nested *t*-test, Means 1.4 and 19.8, SEM 0.4 and 7, *p* < 0.05, *t* = 2.6, df = 4), suggesting that on trials where the cues presented conflicting information, rats “regressed” to the initial rule, even after having completed correct conflict trials during the second block. Importantly, these errors suggest that rats were attending to both rule dimensions after a shift occurred. To determine if one rule modality was represented more than another, we broke down the rule neurons by their preferred rule modality (odor or light). We found no differences in numbers of neurons representing either light or odor rule (37 preferred light rule, 42 preferred odor rule, 2-sample *z*-test to compare sample proportions, *p* = 0.4). In addition, rats showed no difference behavioral whether they started on odor rule and shifted to lights (32.1 trials to switch, SEM 7.9) or started on light rule and shifted to odor rule (36.6 trials to switch, SEM 8.2). Additionally, there was a significant difference of reaction times (time from cue off-set to when rats left the odor port) between compatible and incompatible trial-types (Figure 1E; *t*-test, *p* < 0.001, *t* = 4.0, df = 50, Cohen’s *d* = 0.6, effect size = 0.3). Together, these data demonstrate that rats learned the initial rule in block one, and had difficulty switching to the new rule in block two. Additionally, there was an effect of trial-type on reaction time, demonstrating that rats took longer to make choices when rules were in “conflict” with each other.

**Figure 2:**
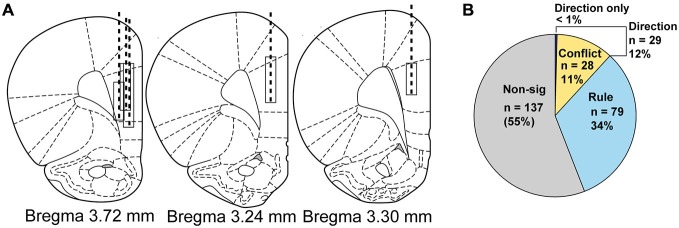
**Electrode implant beginning and final positions, drive paths and classification of neural activity by regression. (A)** Atlas schematics showing electrode wire bundle implant locations (top of box) and location at completion of study (bottom of box) and general drive path (dashed line) for five rats. **(B)** Pie chart showing by number and percentage the breakdown of neural activity types from regression analysis on neural firing, with non-modulated neuron data shown in gray, rule neurons in light blue, conflict neurons in yellow and direction neurons in purple.

We recorded single unit activity from 245 neurons during 51 sessions when rats (*n* = 5, Figure 2A) completed rule shifts. Multiple linear regression analysis of neural firing during cue epoch allowed us to categorize neurons by activity according to different task features, as shown in Figure 2B. Of the 245 neurons, 108 neurons (46%, a significant percentage *χ*^2^ = 1168, *p* < 0.001) were modulated by rule (Figure 2B light blue wedge) or direction. Seventy-nine of those were modulated by rule but not direction or compatibility. Compatibility—whether cues were compatible (i.e., low conflict) or incompatible (high conflict)—was reflected in activity of 28 neurons (Figure 2B, yellow wedge), or 11%, all of which were also modulated by direction. In only 1 neuron was activity modulated by direction, independent from rule and compatibility parameters. Thus, the regression analysis divided neurons into two main groups; (1) neurons whose activity reflected the current rule; (2) neurons whose activity reflected response direction and compatibility of the cues. Though different numbers of neurons were recorded from each rat (Rat 1: 53, Rat 2: 45, Rat 3: 32, Rat 4: 50, Rat 5: 65) the proportional representation of each neural subgroup as identified in the regression analysis was equivalent. *χ*^2^ analysis of the proportion of neurons observed in each subgroup revealed no significant differences in the representation of each animal’s data (*χ*^2^ = 60, *p* = 0.3) (Rule cells: Rat 1: 25%, Rat 2: 40%, Rat 3: 28%, Rat 4: 24%, Rat 5: 42%; Conflict cells: Rat 1: 8%, Rat 2: 9%, Rat 3: 9%, Rat 4: 18%, Rat 5: 13%).

### Rule Encoding in mPFC

**Figure 3:**
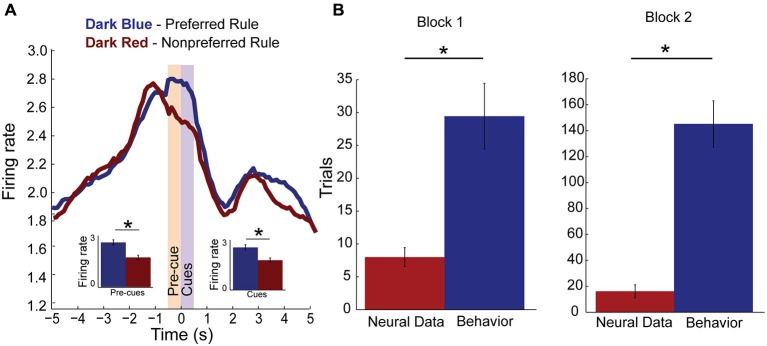
**Rule encoding in the medial Prefrontal Cortex (mPFC). (A)** Population activity of rule neurons, plotted by preferred (dark blue) and non-preferred (dark red) rule blocks centered on cue presentation (time 0). Activity significantly divides during pre-cue epoch (yellow bar, −500–0 ms, inset) and remains higher for one rule over another during the cue epoch (purple bar, 0–500 ms, inset). **(B)** Bar plots comparing when rule neurons first significantly modulate activity in preferred rule block (red bar) and when rats first reached behavioral criterion (blue bar). Change in rule neuron activity precedes behavioral criterion regardless of whether the neuron’s preferred rule was in block one or two. Significance of at least *p* < 0.05 denoted with asterisk *.

Figure 3A plots the neural activity for rule-only neurons for preferred and non-preferred rules, averaged over response direction and compatibility (*n* = 79). For each neuron, “preferred rule” is defined by the rule that elicited the maximal response before averaging. Thus, it is no surprise when averaging activity over all neurons that activity elicited during the preferred rule (dark blue line) is significantly higher than during its non-preferred rule (dark red line) when presented with the cues (purple shaded epoch, *F*_(1,156)_ = 5.33, *p* < 0.05). This result confirms the results of our regression analysis. Interestingly, however, we also see that activity is significantly higher during pre-cue delay before light and odor cues were ever presented (yellow shaded epoch, *F*_(1,156)_ = 4.58, *p* < 0.05). *Post hoc t*-tests supported the ANOVA results for both pre-cue and cue epochs (pre-cue: Means 0.25 and 0.17, SEM, 0.015 and 0.013, *p* < 0.001, *t* = 13.2, df = 78, Cohen’s *d* = 0.7, effect size = 0.32: and cue: Means, 0.24 and 0.17, SEM, 0.015 and 0.013, *p* < 0.001, *t* = 13.4, df = 78, Cohen’s *d* = 0.6, effect size = 0.3) epoch data plotted as inset bar graphs demonstrating that mPFC rule neurons robustly encoded one rule over another, even in anticipation of the cues.

Next we asked how many neurons were modulated during the trial block. To do this we averaged activity over the first 3 trials and compared that firing to a subsequent 3 trial window that slid in 1 trial increments. We found that 34 (43% of rule cells) were modulated within the trial block. Modulation within a rule block occurred with equal regularity in block one (*n* = 19) or block two (*n* = 15). To find if mPFC rule cell encoding was modulated before behavioral changes occurred or if they followed changes to behavior, we found the first trial when the neural activity was significantly different from the start of the preferred block. In addition, we plotted the average trial when those rats reached their behavioral criterion. Figure 3B demonstrates that in block one, a subset of rule-only cells significantly modulated activity well before reaching criterion (*t*-test, *p* < 0.001, *t* = −3.8, df = 6, Cohen’s *d* = −2.2, effect size = −0.74). The same is true for rule-only cells observed in block two (*t*-test, *p* < 0.001, *t* = −9.7, df = 5, Cohen’s *d* = −4, effect size = −1.0). These data suggest that a population of rule-only neurons began to represent the correct rule strategy before the animal’s behavior adjusted appropriately.

**Figure 4:**
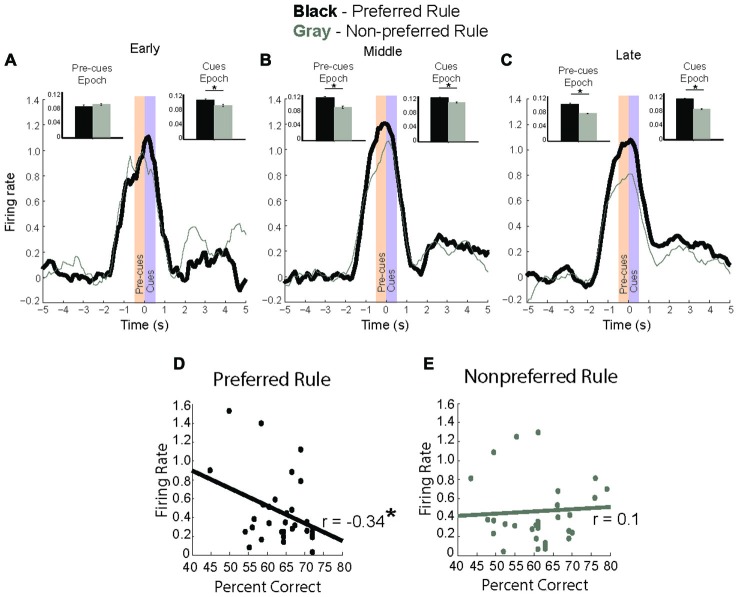
**Rule modulated neurons signal rule change. (A–C)** baseline subtracted neural activity for preferred (black) and non-preferred (gray) rule blocks with inset bar plots comparing neural firing during pre-cue and cue epochs between rule representations. **(A)** In first two trials of a rule block, neuronal activity is significantly higher during cue epoch for preferred rule. **(B)** In the next eight trials, neural activity for preferred rule increases compared to the early trials, and is significantly higher for preferred rule during both the pre-cue epoch and cue epoch. **(C)** In the last ten trials, neural activity for rules has significantly decreased compared to the middle eight, though it remains significantly higher for pre-cue and cue epochs compared to non-preferred rule blocks. **(D)** Correlation of firing rate during the cue epoch with percent correct, showing a significant negative correlation between neural activity and percent correct for the preferred rule block. **(E)** Unlike during preferred rule blocks, firing rate and percent correct for the non-preferred rule block demonstrates no significant correlation. Significance denoted of at least *p* < 0.05 with asterisk *.

To better visualize the time course of rule selectivity within these neurons we plotted the mean firing rate (Figures 4A–C) for these rule-modulated neurons early (first two correct trials), middle (next eight correct) and late (last ten correct) while subtracting the baseline activity for each of those trials and plotting activity during preferred rule (black, thick line) and non-preferred rule (gray, thin line). We chose the first two trials as our early time point because in the analysis above, neurons would significantly change firing within 8–16 trials (Figure 3B). Thus, by examining the first 2 trials of each of 4 trial types we can examine activity preceding the shift, allowing us to determine when changes in neural signals might develop, both during the pre-cue epoch or cue epoch. Inset bar graphs represent the firing rate difference for pre-cue and cue epochs at the different stages in a rule shift (+/− SEM). Early in a block, neural activity of rule modulated neurons was not significantly different during pre-cue epoch (*t*-test, Means 0.63 and 0.41, SEM 0.09 and 0.07, *p* = 0.3, *t* = 1, df = 33, Cohen’s *d* = 0.1, effect size = 0.1) and was significantly higher during the cue epoch (*t*-test, *p* < 0.01, *t* = 4.6, df = 33, Cohen’s *d* = 0.5, effect size = 0.23) for preferred vs. non-preferred rule blocks. In the “middle” phase (trials 3–10), neural activity for the preferred rule block was significantly elevated compared to non-preferred rule block during both the pre-cue epoch (*t*-test, Means 0.5 and 0.4, SEM 0.06 and 0.05, *p* < 0.01, *t* = 3.6, df = 33, Cohen’s *d* = 0.41, effect size = 0.2) and the cue epoch (*t*-test, Means 0.6 and 0.4, SEM 0.08 and 0.76, *p* < 0.01, *t* = 2.8, df = 33, Cohen’s *d* = 0.3, effect size = 0.15). Late in a rule block (last 10 trials), activity for preferred rules was still stronger than non-preferred rules during pre-cue epoch (*t*-test, Means 0.51 and 0.33, SEM 0.07 and 0.05, *p* < 0.001, *t* = 4.0, df = 33, Cohen’s *d* = 0.5, effect size = 0.25) and cue epoch (*t*-test, Means 0.5 and 0.4, SEM 0.06 and 0.06, *p* < 0.01, *t* = 4.2, df = 33, Cohen’s *d* = 0.3, effect size = 0.14).

Interestingly, mPFC rule-only neurons exhibited elevated activity in middle trials, compared to early or late trials. Preferred rule activity during the middle phase was elevated compared to early trials (*t*-test, Means 0.46 and 0.54, SEM 0.04 and 0.03, *p* < 0.001, *t* = 4.5, df = 33, Cohen’s *d* = 0.32, effect size = 0.2) during both pre-cue and cue epochs (*t*-test, Means 0.46 and 0.58, SEM 0.05 and 0.04, *p* < 0.05, *t* = 3.0, df = 33, Cohen’s *d* = 0.4, effect size = 0.2) (black lines, Figures 4A,B). This was not true for non-preferred rule activity, which was unmodulated during pre-cue (*t*-test, Means 0.41 and 0.4, SEM 0.06 and 0.05, *p* = 0.87 *t* = 0.15, df = 33, Cohen’s *d* = 0.02, effect size = 0.01) or for cue activity (*t*-test, Means 0.41 and 0.45, SEM 0.07 and 0.07, *p* = 0.18 *t* = 2.7, df = 33, Cohen’s *d* = 0.17, effect size = 0.1) (Gray lines, Figures 4A,B). Additionally, pre-cue and cue epoch activity were significantly decreased (*t*-test, Means 0.6 and 0.4, SEM 0.07 and 0.05, *p* < 0.01, *t* = 3.0, df = 33, Cohen’s *d* = 0.22, effect size = 0.11) late compared with middle trials in the preferred rule block (black lines, Figures 4B,C). The same was true among non-preferred rule activity, which was significantly decreased on late compared to middle (*t*-test, Means 0.5 and 0.37, SEM 0.06 and 0.05, *p* < 0.01, *t* = 2.7, df = 33, Cohen’s *d* = 0.2, effect size = 0.14) trials (Gray lines, Figures 4B,C). Thus in addition to a divergence between preferred and non-preferred rules, there appears to be a second signal; where activity in the preferred rule increases early in the block and wanes with learning. Increases in firing that take several trials to develop are consistent with previous reports demonstrating that it takes several trials for changes in attention to be engaged after reward prediction errors (RPEs) have been detected. These data suggest that mPFC rule neurons may be important for signaling the need to shift and the appropriate rule.

Because neural data for rule neurons changed over the course of their preferred rule, we hypothesized that the change in rule signal may be related to improved behavioral performance. To this end, we plotted the average neural firing rate during the cue epoch for preferred and non-preferred rule blocks against behavioral performance (percent correct) for the session. Figure 4D demonstrates a significant negative correlation (*r* = −0.34, *p* < 0.05) for rule neurons in the preferred rule block, but not in the non-preferred rule block (Figure 4E) (*r* = 0.1, *p* = 0.8). Together, these data suggest that mPFC rule representations strengthen over the course of the block, as the difference between activity for preferred and non-preferred rules diverges over time.

**Figure 5:**
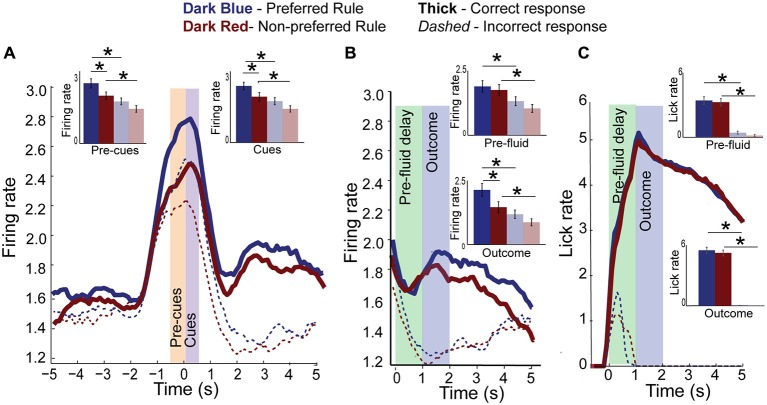
**Rule activity is diminished on error trials. (A)** Population histogram of all rule-modulated neurons by preferred (dark blue) and non-preferred (dark red) rule blocks for correct (solid) and error (dashed) trials with inset bar graphs showing data from pre-cue and cue epochs for correct trials and error trials (faded blue and red bars). Though neural activity is the same as correct trials at baseline, it is significantly reduced during pre-cue and cue epochs. **(B)** Aligning the data from **(A)** to fluid-well entry and plotting pre-fluid delay and reward delivery epochs, we see that neural activity of rule-modulated neurons also reflects trial outcomes, being significantly reduced on error trials and highest during preferred rule correct trials. **(C)** Licking aligned to fluid well entry. Reward is delivered 1 s later on correct trials. Licking did not significantly differ between rules. Significance denoted of at least *p* < 0.05 with asterisk *.

Since activity of rule-modulated neurons on correct trials changes over a block and was correlated with performance, we hypothesized that failure to properly reflect the current rule might underlie erroneous decisions. To test this hypothesis, we plotted activity on error (Figure 5A; thin dashed) and correct (Figure 5A; thick solid) trials during both preferred (Figure 5A; dark blue) and non-preferred (Figure 5A; dark red) rule blocks. Consistent with our hypothesis, we found that activity was diminished on trials during both the pre-cue (*F*_(3,135)_ = 9.67, *p* < 0.001) and the cue epochs (*F*_(3,135)_ = 6.52, *p* < 0.001), with *post hoc t*-test revealing significant differences between preferred rule and non-preferred rule correct and error activity during pre-cue (Means 0.3 and 0.23, SEM, 0.02 and 0.02, *p* < 0.01, *t* = 4, df = 33, Cohen’s *d* = 0.6, effect size = 0.3, inset) and cue epoch (Means 0.3 and 0.22, SEM, 0.02 and 0.02, *p* < 0.001, *t* = 3.4, df = 33, Cohen’s *d* = 0.6, effect size = 0.3), respectively and lower activity for non-preferred errors than preferred rule errors (Means 0.3 and 0.23, SEM 0.02 and 0.2, *p* < 0.05, *t* = 2.4, df = 33, Cohen’s *d* = 0.5, effect size = 0.24 light blue vs. light red bars, inset) during cue epoch. More importantly, on errors, the difference between preferred rule and non-preferred rule was not significant during either epoch (pre-cue: *t*-test, Means 0.23 and 0.22, SEM 0.01 and 0.01, *p* = 0.3, *t* = 0.9, df = 33, Cohen’s *d* = 0.14, effect size = 0.1, inset; cue *t*-test, Means 0.22 and 0.2, SEM 0.02 and 0.02, *p* = 0.2, *t* = 1.4, df = 33, Cohen’s *d* = 0.21 effect size = 0.11, inset). These data suggest that when activity in mPFC was low and rule selectivity was reduced, rats incorrectly followed the wrong rule.

Surprisingly, neural activity in this population of neurons was also modulated during the outcome phase. Figure 5B shows activity of the same neurons aligned to fluid-well entry, and plots activity through the 1000 ms pre-fluid delay (green bar) to reward delivery (blue bar). Neural activity of rule neurons on both preferred and non-preferred rule blocks was significantly lower after erroneous choices during both the pre-fluid delay (*F*_(3,135)_ = 4.03, *p* < 0.01) and outcome epoch (*F*_(3,135)_ = 7.41, *p* < 0.001) compared to correct choices and compared to baseline (*F*_(1,135)_ = 5.33, *p* < 0.05). *Post hoc t*-tests identified significant differences between both pre-fluid delay and outcome epochs for correct vs. error trials (preferred rule correct vs. error, Means 0.2 and 0.13, SEM 0.02 and 0.02, *p* < 0.001, *t* = 3, df = 33, Cohen’s *d* = 0.5 effect size = 0.24, non-preferred rule, Means 0.21 and 0.12, SEM 0.03 and 0.02, *p* < 0.001, *t* = 3.3, df = 33, Cohen’s *d* = 0.65, effect size = 0.31, inset). Significant differences also exist during the outcome epoch for preferred vs. non-preferred rules correct trials (preferred rule, Means 0.21 and 0.15, SEM 0.3 and 0.2, *p* < 0.05, *t* = 4.8, df = 33, Cohen’s *d* = 0.77, effect size = 0.36, non-preferred rule, Means 0.15 and 0.12, SEM 0.02 and 0.02, *p* < 0.05, *t* = 3.5, df = 33, Cohen’s *d* = 0.55, effect size = 0.3, inset), but not on error trials (Means 0.11 and 0.09, SEM 0.02 and 0.02, *p* = 0.8 *t* = 0.42, df = 33, Cohen’s *d* = 0.12, effect size = 0.04, inset) or during pre-fluid delay epoch (Means 0.19 and 0.18, SEM 0.023 and 0.02, *p* = 0.2, *t* = 0.6, df = 33, Cohen’s *d* = 0.11, effect size = 0.1 and Means 0.13 and 0.01, SEM 0.02 and 0.02, *p* = 0.9, *t* = 0.4, df = 33, Cohen’s *d* = 0.04, effect size = 0.1, correct and error trials, respectively, inset bar graph).

It might be argued that activity during both the pre-fluid delay and outcome epochs reflects differences in licking. However, this interpretation does not hold for several reasons. First, we will show that rats do not lick differently during rewards delivered in different rule blocks. Second, licking rapidly increased immediately after entering the fluid well, while neural activity declined. Increasing licking activity was only observed during anticipation of reward delivery, as lick rates slowed once the fluid was delivered. Furthermore, on error trials activity rapidly decreased during the pre-fluid delay even through rats were licking during this period. Finally, on error trials, firing rates dropped below baseline during the outcome epoch. Thus, during both baseline and outcome epochs there was no licking, but activity significantly differed.

These arguments are supported by Figure 5C which plots lick activity, by photobeam breaks in the fluid well. There was a significant main effect of correctness (*F*_(3,135)_ = 45.85, *p* < 0.001) during the pre-fluid delay epoch. *Post hoc* *t*-tests demonstrate that rats begin licking on correct trials before reward delivery equally for both preferred and non-preferred rules (*p* = 0.5). Rats also lick throughout the pre-fluid delay on error trials, despite receiving feedback that their choice was incorrect (house lights turn off upon wrong fluid-well entry), though significantly less than on correct trials (*p* < 0.001). The same is true of the outcome epoch, where there was a significant main effect of correctness (*F*_(3,135)_ = 212, *p* < 0.001), where error responses elicited significantly decreased neural activity compared to correct responses for preferred or non-preferred rules (*p* < 0.001, = 17.8, df = 33, Cohen’s *d* = 4.3, effect size = 0.91). These data support the idea that differences in firing between correct vs. incorrect and preferred vs. non-preferred rules does not reflect differences in licking behavior during the trial feedback. Furthermore, increased licking during the pre-fluid delay on error trials supports the notion that rats, albeit to a lesser degree, still anticipated reward on those trials, suggesting that decreased firing observed with erroneous outcomes might reflect a worse-than-expected outcome.

### Conflict Encoding in mPFC

**Figure 6:**
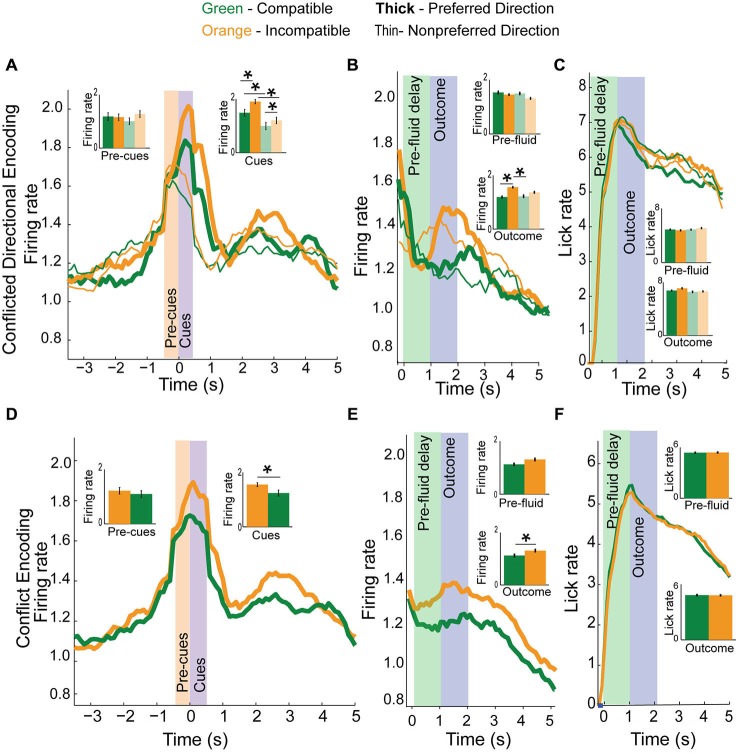
**Conflict and direction activity in the mPFC. (A)** Top panel plots direction neurons based on preferred direction (thick) and non-preferred direction (thin) and by trial-type, compatible (green) and incompatible (orange) with inset bar graphs showing data during both pre-cue and cue epochs for correct and error (faded green and yellow) trials. While direction neurons were more active for one direction over another, activity in these neurons was more active when directional cues were in conflict than when they were compatible with each other. **(B)** Direction and conflict data demonstrates elevated activity for preferred direction incompatible trials during the outcome epoch, but not pre-fluid epoch. **(C)** Plots licking data demonstrating no difference in lick rate between trial-types. **(D)** Collapsing the data from **(A)** into compatible or incompatible trials, we see that directional neurons fire more for incompatible trial-types than for compatible trial-types when presented with cue information. **(E)** Activity plotted by compatibility averaged across direction during the pre-fluid and outcome epochs demonstrates a significant elevation of activity on correct incompatible trial-types compared to compatible trial-types but not during the pre-fluid epoch. **(F)** Plots licking rate by compatibility, and demonstrates no difference in lick rate between compatible or incompatible trial-types in either the pre-fluid or outcome epochs. Significance denoted of at least *p* < 0.05 with asterisk *.

We observed 29 (12%) of neurons which encoded direction in our task, 28 of which also reflected the conflict inherent in the different trial-types. Figure 6A illustrates the average neural activity for direction neurons, broken down into preferred direction (thick lines), non-preferred direction (thin lines) and by trial type, with cues in compatible (green) or incompatible directions (yellow). There was a significant effect of trial-type and direction (*F*_(3,115)_ = 11.04, *p* < 0.001) of firing during the cue epoch, but not during the pre-cue epoch (*F*_(3,115)_ = 0.74, *p* = 0.53). *Post hoc t*-tests of firing during the cue epoch revealed elevated firing for incompatible trials in both the preferred and non-preferred directions (Means 0.14 and 0.2, SEM 0.01 and 0.01, *p* < 0.05, *t* = 2.2, df = 28, Cohen’s *d* = 0.5, effect size = 0.24) compared to compatible trial-types (inset bar plots; Figure 6A, Yellow vs. Green). This effect was true when a correct response was in the neuron’s preferred direction (Figure 6A thick Yellow vs. thick Green and inset, Means 0.14 and 0.19, SEM 0.013 and 0.008, *t*-test, *p* < 0.05, *t* = 2.3, df = 28, Cohen’s *d* = 0.52, effect size = 0.25) and the non-preferred direction (Thin yellow vs. thin green and inset, *t*-test, Means 0.09 and 0.12, SEM 0.01 and 0.012, *p* < 0.05, *t* = 2.2, df = 28, Cohen’s *d* = 0.53, effect size = 0.3). To investigate whether activity of direction neurons reflected outcomes, we aligned the data to fluid-well entry (Figure 6B), allowing comparison of pre-fluid and outcome epochs. There was no significant difference between directions or compatibility during the pre-fluid delay (*F*_(3,115)_ = 0.25, *p* = 0.9) but there was during the outcome epoch (*F*_(3,115)_ = 4.29, *p* < 0.01). *Post hoc t*-tests revealed a significant difference of activity on preferred direction incompatible trials during the outcome epoch, compared to preferred direction compatible trials (Means 0.2 and 0.14, SEM 0.01 and 0.01, *p* < 0.001, *t* = −2.04, df = 27, cohen’s *d* = −0.5, effect size = −0.24) and compatible non-preferred direction (Means 0.17 and 0.12, SEM 0.02 and 0.01, *p* < 0.001, *t* = −4.2, df = 28, Cohen’s *d* = −0.2, effect size = −0.2). There were no differences in how the rats licked (Figure 6C) during the pre-fluid epoch (*F*_(3,115)_ = 1.78, *p* = 0.2) or the outcome epoch (*F*_(3,115)_ = 0.53, *p* = 0.7) suggesting that differences in firing between compatible and incompatible trials cannot merely reflect differences in licking.

To further illustrate differences in neural activity for incompatible and compatible trials, we collapsed across directions and plotted neural activity for compatible and incompatible trial-types. Figure 6D shows the same data as 6a, but collapsed across directions to display compatible vs. incompatible activity. There was no significant difference in neural activity between compatible or incompatible trial-types during the pre-cue epoch (Means 0.12 and 0.11, SEM 0.01 and 0.01, *t*-test, *p* = 0.14, *t* = 1.5, df = 28, Cohen’s *d* = 0.1, effect size = 0.1, inset), and activity was significantly elevated on incompatible trial-types during the cue epoch (Means 0.16 and 0.12, SEM 0.007 and 0.01, *t*-test, *p* < 0.01, *t* = 4.0, df = 28, Cohen’s *d* = 0.44, effect size = 0.22, inset). Plotting compatibility during trial-outcomes (Figure 6E), we observed that there was no significant difference in neural activity during the pre-fluid delay (Means 0.12 and 0.13, SEM 0.03 and 0.02, *t*-test, *p* = 0.2, *t* = −1.3, df = 28, Cohen’s *d* = −0.14, effect size = −0.1, inset) but there was a significant difference in neural activity during the outcome epoch (Means 0.12 and 0.15, SEM 0.01 and 0.01, *t*-test, *p* < 0.01, *t* = −4.2, df = 27, cohen’s *d* = −0.21, effect size = −0.1, inset). There was no difference in lick rate (Figure 6F) during either the pre-fluid period (Means 4.8 and 4.8, SEM 0.3 and 0.3, *t*-test, *p* = 0.8, *t* = 0.2, df = 27, Cohen’s *d* = 0.02, effect size = 0.01, inset) or the outcome epoch (Means 6.6 and 6.7, SEM 0.3 and 0.3, *t*-test, *p* = 0.9, *t* = 0.9, df = 27, Cohen’s *d* = 0.03, effect size = 0.02, inset).

**Figure 7:**
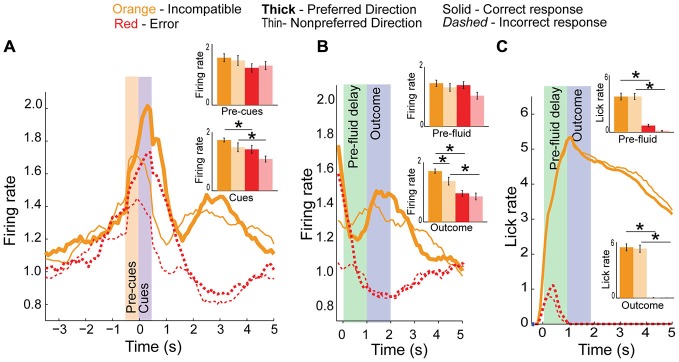
**Conflict activity on error trials. (A)** Population histogram of conflict neurons on incompatible trial-types (orange) plotted by direction (full color are preferred direction, faded color are non-preferred directions in bar graph insets) showing decreased neural activity on error trials (dashed red or faded red for inset bar graphs) during the cue epoch. **(B)** Aligning data to fluid-well entry demonstrates a significant reduction in activity during pre-fluid delay and after reward should have been delivered, suggesting these neurons also represent the outcome of the trials. **(C)** Licking did not significantly differ between directions. Significance denoted of at least *p* < 0.05 with asterisk *.

Increased firing on incompatible trials might be necessary to resolve high conflict when two rules oppose each other. To test this hypothesis we compared firing on error trials to correct responses made in the same response direction (Figure 7A). We observed a significant main effect of correctness during the cue epoch (*F*_(3,115)_ = 4.25, *p* < 0.01) but not during the pre-cue epoch (*F*_(3,115)_ = 0.97, *p* = 0.4). *Post hoc t*-tests revealed a significant reduction in activity for error trials, compared to correct trial activity in both the preferred or non-preferred directions (*p* < 0.05, *t* = 2.3, df = 27, Cohen’s *d* = 0.52, effect size = 0.25, inset bar graphs) during the cue epoch. Thus, when firing was low, rats tended to make errors.

Neural activity of the same neurons is aligned to fluid well entry is illustrated in Figure 7B. Activity was not significantly different between correct and incorrect trials during the pre-fluid delay (*F*_(3,115)_ = 2.1, *p* = 0.1) though it was significantly different during the outcome epoch (*F*_(3,115)_ = 5, *p* < 0.01) with *post hoc t*-test revealing significant differences between preferred and non-preferred direction correct and error activity during the outcome epoch (Means 0.24 and 0.3, SEM 0.02 and 0.02, *p* < 0.001, *t* = 6.9, df = 27, Cohen’s *d* = 1.5, effect size = 0.6 and Means 0.16 and 0.1, SEM 0.02 and 0.01, *p* < 0.01, *t* = 3.3, df = 27, Cohen’s *d* = 0.72, effect size = 0.34, respectively, inset). In addition to signaling whether or not reward was delivered, activity during the outcome epoch was significantly higher in the preferred direction (as defined during cue epoch) relative to the non-preferred direction (Means 0.18 and 0.12, SEM 0.02 and 0.02, *t*-test, inset, *p* < 0.05, *t* = 2.1, df = 27, Cohen’s *d* = 0.54, effect size = 0.26). As highlighted above, these differences cannot reflect differences in licking; licking was not different between preferred and non-preferred directions and neural activity during the outcome epoch was reduced relative to the baseline epoch Figure 7C.

## Discussion

Numerous studies have supported a critical role for the mPFC in managing the shift between rule-based responses (Dias et al., 1996a,b; Birrell and Brown, 2000; Colacicco et al., 2002; Bissonette et al., 2008). These studies have investigated rule shifting across a number of different sensory modalities and response strategies including shifts from cued to egocentric responses in maze tasks (Ragozzino et al., 1999a,b), operant tasks (Floresco et al., 2008; Durstewitz et al., 2010) and shifts between different cued responses (digging tasks) (Birrell and Brown, 2000; Colacicco et al., 2002; Bissonette et al., 2008). Across nearly all of these tasks, rats were able to respond reliably to rule-based response options, but showed impairment when contingencies shift so that previously reliable response options are no longer predictive of a rewarded outcome. Critically, nearly any perturbation to the prefrontal cortex has elicited these deficits, including lesions studies (Dias et al., 1996b; Birrell and Brown, 2000; Bissonette et al., 2008), pharmacological inactivation (Stefani et al., 2003; Floresco et al., 2008) and pharmacological interventions focusing on the role of dopamine (DA; Ragozzino, 2002; Floresco et al., 2006), GluN2B (Stefani and Moghaddam, 2006; Dalton et al., 2011; Brigman et al., 2013; Marquardt et al., 2014) and norepinephrine (Tait et al., 2007), genetic manipulations (Brigman et al., 2013; Bissonette et al., 2014) and recently, optogenetically (Cho et al., 2015).

Though there is a wealth of research demonstrating a critical role for mPFC in mediating rule shifting, there is a dearth of recording studies in rats, identifying how the prefrontal cortex may be accomplishing this task. Primate studies (White and Wise, 1999; Wallis et al., 2001; Bunge et al., 2003; Muhammad et al., 2006; Cromer et al., 2010; Antzoulatos and Miller, 2011) dominate the field, with only one rat study (Durstewitz et al., 2010) suggesting that rat prefrontal neurons switch encoding as ensembles, together representing a rule. In our task, we were able to counterbalance directional responding evenly across all rule blocks, and are thus able to dissociate neural correlates of direction, conflict and rules. Further, by presenting two separate external cues, we control for the possibility that neurons representing a directional response are active during the epoch once cues are presented, and not an unknown time before. Our data support these previous results, and further demonstrates that individual neurons in the rat mPFC more strongly encode one rule block over another. Additionally, our data suggest that not all neurons are involved in abstract rule encoding. In our task, a subset of mPFC neurons was directionally tuned, such that activity reflected a preferred response direction. Notably, these neurons not only represented the response direction, but activity of these neurons reflected heightened conflict on challenging trial-types (i.e., conflict between two rules). Thus, while activity of some neurons represented the current rule block, a separate population of neurons were more active on incompatible trial-types, when more attentional resources were needed in order to decide which response option was appropriate. All of these electrophysiological comparisons are possible because of the rigorous nature of this behavioral task.

Neurons in the mPFC which were selective for one rule over another were active not only during cue presentation, but also during the pre-cue epoch. This pattern of activity differed from the conflict-neuron population, where activity could only diverge once the directional information was presented. This pattern of activity suggests that rule neurons may be calling up the preferred rule representation and holding it online prior to the instruction to guide downstream areas to select particular actions.

These data are suggestive that the critical role for mPFC rule encoding is separate from the role for the conflict signaling neural populations. Medial PFC rule neurons may signal the shift between rules which quickly passes this information off to downstream areas such as the dorsal striatum to handle the specific behavioral responses. If this is true, neurons in dorsal striatum might also reflect some measure of abstract rule representation, and rule encoding should increase just as mPFC rule signaling decreases. Such a signal would allow mPFC to influence specific action selection in dorsal striatum, and would provide the necessary “shift” signal to striatal neurons. Indeed, two disconnection studies and a recent electrophysiological study support this notion. Functional disconnection of the ventral striatum (nucleus accumbens) from mPFC with bupivacaine in a strategy shifting task led to a significant increase in number of trials required to shift response strategies in a plus maze task (Block et al., 2007). Recently, disconnection of the prelimbic region of rat mPFC from the dorsomedial striatum was shown to disrupt cue-guided behavioral switching (Baker and Ragozzino, 2014). Importantly, this study demonstrated that contralateral disconnection of prelimbic cortex and dorsomedial striatum did not impact a cued-association task which did not require switching between rules, suggesting an important role in task switching for this circuit.

Another recent study also demonstrated a role for dorsomedial striatum in set-shifting, while identifying robust neural correlates of rule encoding in dorsal striatal neurons. Dorsal striatal direction neurons reflected directional conflict, though interestingly in opposite sign to the results observed in mPFC in this manuscript. Additionally, the directional conflict signal in mDS was resolved as animals improved performance, demonstrating a possible neural mechanism by which rules guiding behavior impact action selection (Bissonette and Roesch, 2015). Recent research has also supported a role for dorsal striatum in mediating more abstract aspects of cognition, potentially as a site for disruption due to psychiatric illness (Ragozzino, 2007; Miller et al., 2015). Perhaps mPFC conflict neuron activity levels reflect the need for attention on especially salient and challenging trial-types, providing an alerting function for other neural regions to dedicate more resources to resolving the trial at hand.

Interestingly, the selectivity of rule encoding neurons took several trials to develop and was stronger earlier in the block compared to later. Riding on top of this signal was a general increase in activity that was strongest early in the rule block, and waned over the trial block. This was true in both the preferred and non-preferred rule blocks, though activity was more robust in the preferred rule block, and improvement in behavioral success was correlated with decreasing neural firing in a neuron’s preferred rule block. Changing rule encoding has been observed before Durstewitz et al. (2010), where prefrontal neural ensembles reorganized prior to rats shifting behavior. We observed the same phenomenon among our rule neurons, which rapidly modulated their neural activity to change between different rule states. In fact, the change in activity levels occurred well before rats reached criteria, and even preceded the 20-trial window in which rats reached behavioral criteria. These data imply that mPFC rule-neurons rapidly modify their activity levels to quickly reflect the changed contingency, and that behavioral adaptation may rely upon downstream areas receiving, recognizing, and storing this modified mPFC activity state.

A separate population of neurons was responsible for encoding not only the response direction, but also for representing the inherent conflict within the task. That is, directionally tuned neurons were more active on incompatible trial-types. These trial-types presumably require additional attentional resources to respond correctly, and increased directional signaling on incompatible trials may reflect the need for additional attentional resources. Such a signal may reflect mPFC’s role in attentional tasks, especially the role for preparatory attention (Totah et al., 2012). In this case, attention for specific stimulus-responses may reflect not just the stimulus-response aspects of well-trained rats (Corbit and Balleine, 2003) but also the increased attention necessary to successfully complete more challenging trial-types. It may be the role of a subset of mPFC neurons to report the occurrence of an incongruous trial to downstream areas where potential directional responses may be more rigorously evaluated.

Across the board, neural activity during the decision period was diminished when rats made erroneous choices. Rule encoding in either the preferred or non-preferred rule blocks was diminished on error trials, compared to correct response outcomes. This pattern was also observed for the neurons modulated by the degree of conflict; cue-evoked activity was diminished prior to errors made in both the preferred and non-preferred directions. These data suggest that, when mPFC neurons are not actively engaged during task performance and do not accurately represent the correct rule block, or signal the presence of high conflict, rats were more likely to make an error.

Remarkably, both conflict neurons and rule neurons also were active during outcome epochs. Prefrontal cortical neurons are known to multiplex different forms of information across categories (Rainer et al., 1999; Cromer et al., 2010), and several studies have reported prediction error type responses in mPFC and ACC (Brown and Braver, 2005; Totah et al., 2009; Alexander and Brown, 2011; Bryden et al., 2011; Hayden et al., 2011). Activity during the outcome phase of this task represents a potential feedback mechanism by which mPFC neurons may modulate activity patterns based on outcomes and what preceded them (Laubach et al., 2015). Such signals may arise and/or inform ventral tegmental area (VTA) DA neurons whose activity also reflects RPEs (Schultz and Dickinson, 2000; Waelti et al., 2001; Roesch et al., 2007; Glimcher, 2011). This theory is consistent with anatomy showing that mPFC and VTA are reciprocally connected via mesocortical DA projections from VTA to mPFC (Gabbott et al., 2005; Bjorklund and Dunnett, 2007; Hoover and Vertes, 2007), and cortico-tegmental projections back to VTA (Carr and Sesack, 1996; Vertes, 2004).

Importantly, and unlike DA neurons, activity of mPFC reflects more than simple signed prediction errors. In neurons modulated by conflict and rule during the decision period, we observed higher activity for incompatible over compatible responses, and preferred over non-preferred rules, respectively. Elevated firing during high conflict (incompatible) trials might reflect unexpected reward delivery. That is, on incompatible trials, rats might not expect reward as strongly, thus, when it was delivered, it was surprising and elicited a strong positive prediction error. Although this theory is plausible, it is not consistent with the fact that reward predictions, as measured by anticipatory licking, did not significantly differ between correct compatible and incompatible trials. Unlike conflict signals, neurons that carried information about the current rule during the outcome phase cannot be readily explained in terms of unexpected reward delivery. In addition, rats did not lick differently during odor compared to light rule trial blocks. Taken together, these data suggest that single neurons in mPFC are informing downstream regions what direction was chosen, what rule was being followed and the degree of conflict associated with making that decision. Specifically, these results might be signaled to mDS which has been shown to signal action specific RPEs (Stalnaker et al., 2012).

In conclusion, this report is among the first to demonstrate neural correlates of distinct rules in rodent mPFC. Because of the controlled nature of this task, we were also able to separate direction from conflict encoding. Further, because rats were required to maintain head position at the central nosepoke we can clearly dissociate neural correlates related to rule encoding during the pre-cue and cue epochs as opposed to signals related to body position and already planned actions, which were only encoded after cues were presented. Importantly, these results suggest that neural representations of rules in mPFC are not directionally tuned and are strongest earlier in rule blocks when rules need to be distinguished. Other neurons participate in signaling the correct direction. In addition these neurons fire more strongly on high conflict trials, when the two rules opposed each other. Finally, all these task parameters—rule, conflict, and direction—were reflected after the decision, during the outcome phase of the task. Attentional set-shifting is complicated and requires several cognitive control functions that govern which rule should be followed, how much attention is necessary to shift and override competing responses, and whether or not the response that was just made was correct, difficult, and consistent with current rules. Remarkably, mPFC participated in all of these functions during performance of our set-shifting task.

## Author Contributions

G.B.B. and M.R. conceived the experiments, G.B.B. performed the experiments and analysis, and G.B.B. and M.R. wrote the manuscript.

## Funding

We would like to thank our funding source DA031695 (MRR).

## Conflict of Interest Statement

The authors declare that the research was conducted in the absence of any commercial or financial relationships that could be construed as a potential conflict of interest.
